# Endoscopic grasping forceps rescue retrieval of a migrated biliary stent using a sheath-assisted technique

**DOI:** 10.1055/a-2873-7737

**Published:** 2026-06-01

**Authors:** Kenji Urakabe, Akihisa Kato, Akihisa Adachi, Yusuke Kito, Tadashi Toyohara, Yasuki Hori, Michihiro Yoshida

**Affiliations:** 1 Department of Gastroenterology and MetabolismNagoya City University Graduate School of Medical SciencesNagoyaJapan

Proximal migration of biliary plastic stents occasionally occurs and can be difficult to manage, particularly when the stent migrates toward the hepatic hilum or peripheral bile ducts 1 2 . Various retrieval devices – including extraction balloons, basket catheters, snares, and biopsy forceps – are commonly used; however, secure engagement is often difficult 3 4 . Although grasping forceps provide reliable capture of a migrated stent, their use in deeply migrated cases is limited because the rigid tip makes safe advancement across biliary strictures difficult. Consequently, effective stent capture with grasping forceps is often unfeasible in complex anatomy. We report a case in which a large-caliber endoscopic sheath enabled the safe insertion of grasping forceps beyond the stricture, allowing secure retrieval of a proximally migrated stent.

**Fig. 1:**
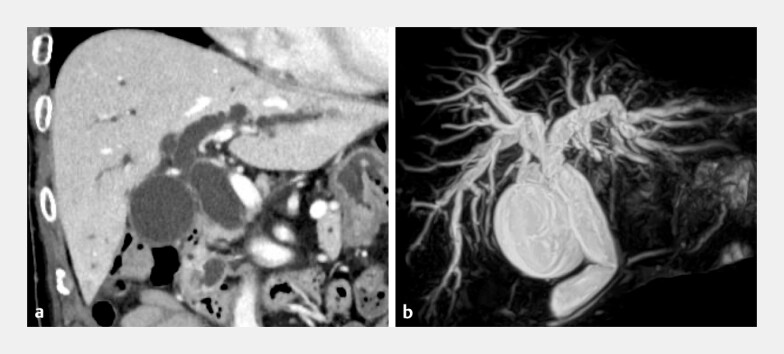
**a** Contrast-enhanced CT demonstrating a 10-mm enhancing nodule at the ampulla of Vater. **b** Magnetic resonance cholangiopancreatography (MRCP) demonstrating diffuse biliary dilatation at initial presentation. CT, computed tomography.

**Fig. 2:**
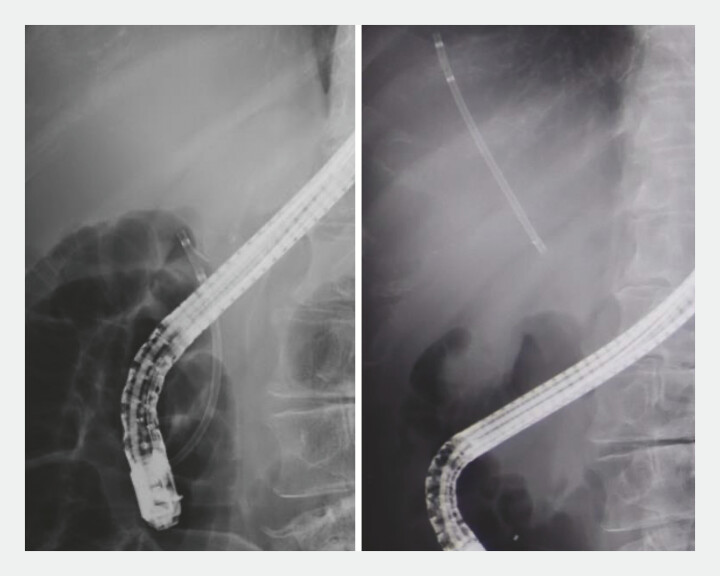
A fluoroscopic image showing a biliary plastic stent deeply migrated into the intrahepatic bile duct.

**Fig. 3:**
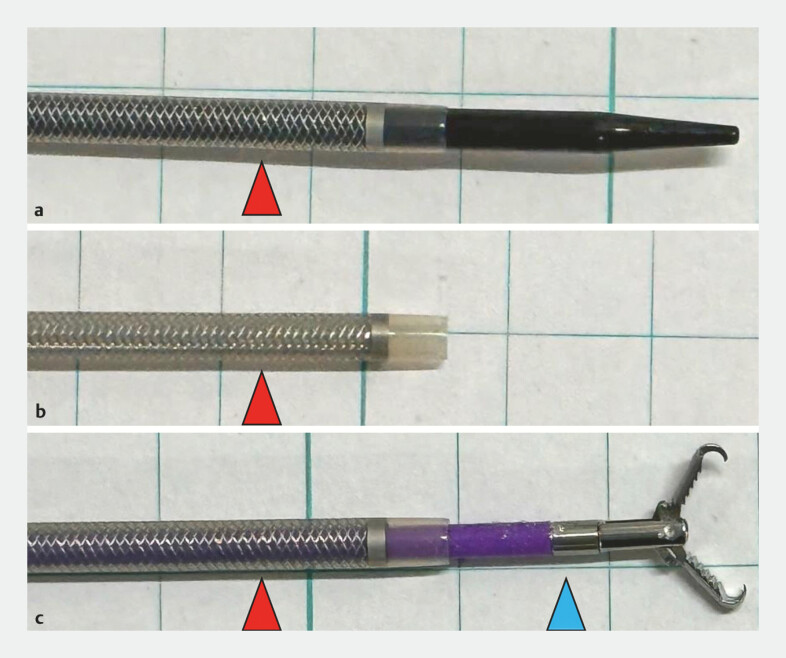
**a** Appearance of the modified large-caliber EndoSheather with the outer sheath and inner catheter assembled, compatible with a 0.025-inch guidewire (Piolax, Inc., Yokohama, Japan). **b** An outer sheath of the EndoSheather (outer diameter: 10 Fr and inner diameter 8.4 Fr). **c** Grasping forceps (2.3-mm diameter; AGS MedTech, Hangzhou, China) inserted into the outer sheath of the EndoSheather. Blue arrowhead: grasping forceps; red arrowhead: a large-caliber endoscopic sheath.

**Fig. 4:**
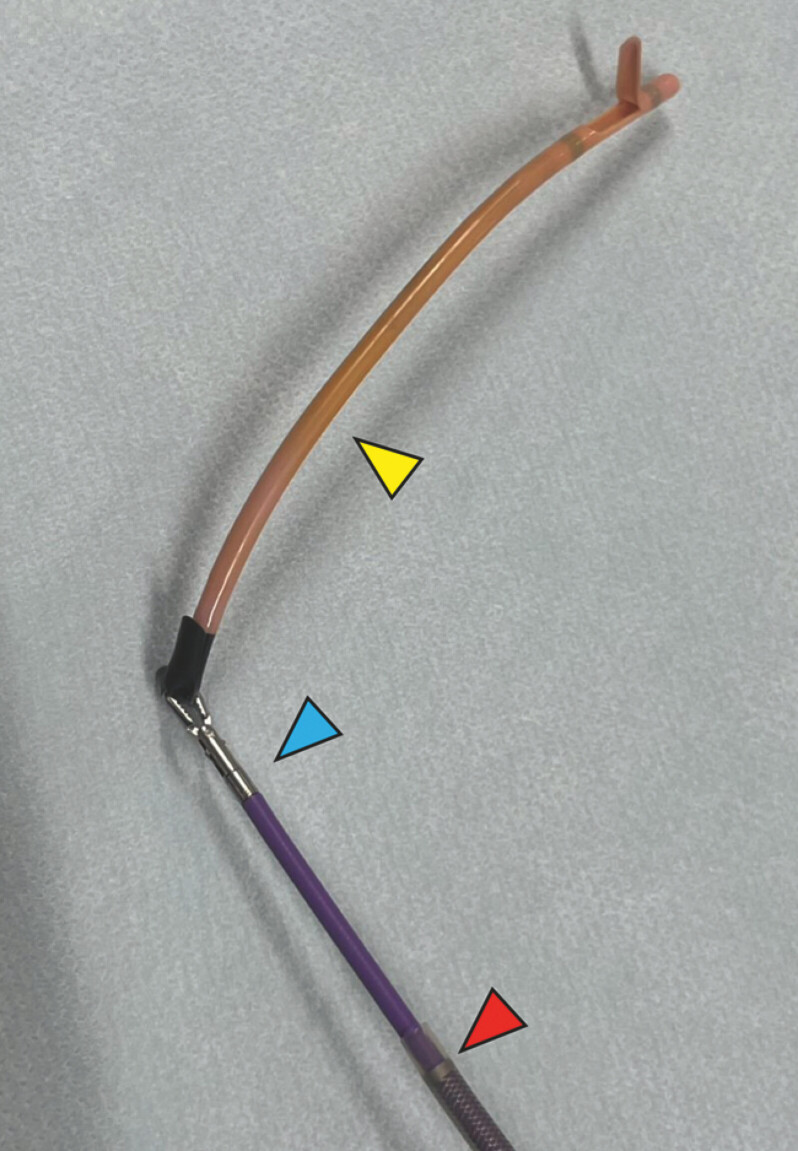
Grasping forceps introduced through the large-caliber endoscopic sheath securely grasping and retrieving the migrated biliary stent. Yellow arrowhead: a migrated stent; blue arrowhead: grasping forceps; red arrowhead: a large-caliber endoscopic sheath.

**Fig. 5:**
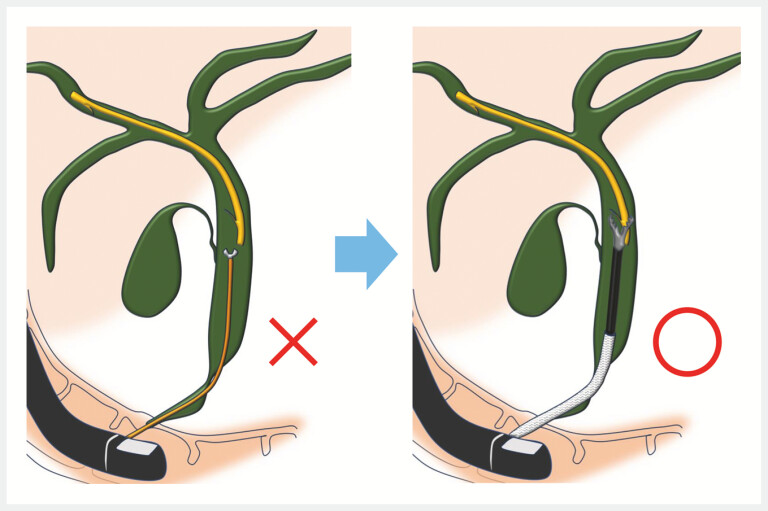
Biopsy forceps could not securely grasp the stent. In contrast, the large-caliber endoscopic sheath allowed the safe delivery of grasping forceps across the stricture to the target site, enabling easy and secure capture of the migrated stent. This technique eliminates the need for cannulation alongside the guidewire, thereby minimizing papillary manipulation and potentially reducing procedure-related complications such as pancreatitis or bleeding.

A 78-year-old man with obstructive jaundice was diagnosed with ampullary adenoma ( Fig. 1 ). An 8.5-Fr plastic stent was placed for biliary drainage, resulting in temporary improvement. One month later, he developed acute cholangitis due to proximal migration of the stent toward the hepatic hilum ( Fig. 2 ). After guidewire placement, tumor involvement prevented biliary access with biopsy forceps. Therefore, a large-caliber endoscopic sheath ( Fig. 3 , 5 ) was advanced over the guidewire, smoothly crossing the stricture and reaching distal to the migrated stent. After the removal of the inner catheter, grasping forceps were introduced through the outer sheath. Under fluoroscopic guidance, the stent was grasped and retrieved into the duodenum without adverse events ( Fig. 4 , Fig. 5 ). A new biliary stent was placed, resulting in clinical improvement ( Video 1 ).

Removal of a migrated biliary plastic stent using a novel large-caliber endoscopic sheath with the sheath-assisted insertion of grasping forceps.Video 1

This case demonstrates that the sheath-assisted insertion of grasping forceps using a large-caliber endoscopic sheath is a safe and effective option for the removal of deeply migrated biliary stents when conventional device advancement is difficult. Advancement over a guidewire allows precise delivery to the target site while minimizing papillary manipulation and potentially reducing complications such as pancreatitis or bleeding.

Endoscopy_UCTN_Code_TTT_1AR_2AZ
